# Correlation between admission hypoalbuminemia and postoperative urinary tract infections in elderly hip fracture patients

**DOI:** 10.1186/s13018-023-04274-7

**Published:** 2023-10-14

**Authors:** Wei Yao, Wanyun Tang, Wei Wang, Qiaomei Lv, Wenbo Ding

**Affiliations:** 1 Department of Orthopedics, Dandong Central Hospital, China Medical University, No. 338 Jinshan Street, Zhenxing District, Dandong, 118002 Liaoning Province People’s Republic of China; 2 Department of Oncology, Dandong Central Hospital, China Medical University, Dandong, People’s Republic of China

**Keywords:** Hip fracture, Elderly, Hypoalbuminemia, Urinary tract infections, Risk factors

## Abstract

**Purpose:**

This study aimed to evaluate the correlation between hypoalbuminemia upon admission and the incidence of postoperative urinary tract infections (UTIs) in elderly patients with hip fractures.

**Methods:**

A retrospective analysis was performed on the medical records of elderly patients who underwent surgical treatment for hip fractures at a level I trauma center from 2013 to 2023. Serum albumin levels were measured upon admission, and hypoalbuminemia was defined as a total albumin level < 35 g/L. Multivariable logistic regression and propensity score matching analysis were utilized to control and reduce potential confounding factors, aiming to obtain adjusted odds ratios (ORs) and 95% confidence intervals (CI) for UTIs to determine the strength of the association.

**Results:**

This observational cohort study included 1279 patients, among whom 298 (23.3%) developed UTIs. Patients with albumin levels < 35 g/L had significantly greater odds of developing UTIs compared to those with albumin levels ≥ 35 g/L (OR 1.86, 95% CI 1.28–2.70). Further analysis, dividing albumin levels into quartiles, demonstrated that patients in the *Q*2 group (38.0–40.9 g/L; OR 1.38, 95% CI 0.88–2.17), *Q*3 group (35.0–37.9 g/L; OR 1.69, 95% CI 1.06–2.71), and *Q*4 group (15.3–34.9 g/L; OR 2.67, 95% CI 1.61–4.43) had notably higher odds of developing UTIs compared to those in the *Q*1 group (41.0–52.0 g/L).

**Conclusions:**

The presence of hypoalbuminemia upon admission in elderly patients undergoing hip fracture surgery is strongly correlated with the occurrence of postoperative UTIs. Furthermore, this association exhibits a clear dose–response relationship.

**Supplementary Information:**

The online version contains supplementary material available at 10.1186/s13018-023-04274-7.

## Introduction

Hip fracture poses a significant health risk for the elderly, and its incidence escalates markedly with age [1, 2]. Projections indicate that global annual hip fracture cases will surge from 1.7 million in 1990 to 6.3 million by 2050 [3, 4]. This not only places a substantial economic burden but also demonstrates a close correlation with postoperative complications, heart failure, and mortality within 30 days [5–7]. In reality, the cumulative mortality rate within one year after surgery can range between 20 and 30% [8–10].

Serum albumin is widely recognized as a crucial indicator for evaluating the nutritional status of patients, as its decreased levels often signify malnutrition [11–13]. Hypoalbuminemia (serum albumin < 35 g/L) is more prevalent among elderly patients with preoperative hip fractures and has been strongly associated with various postoperative complications and early mortality [14–17]. There are several factors contributing to hypoalbuminemia, including chronic diseases, inflammatory responses, and insulin resistance [11, 18, 19]. These physiological and pathological processes can exacerbate inadequate nutritional intake, further compromising the body's recovery and increasing the risk of infections [20–22].

Considering these factors, the aim of this study is to examine the correlation between hypoalbuminemia in elderly patients with hip fractures and the occurrence of postoperative urinary tract infections (UTIs). UTIs are the most prevalent bacterial infections among the elderly and frequently lead to bacteremia/sepsis, particularly in cases involving indwelling urinary catheters [23–25]. The use of indwelling catheters following standardized perioperative management has become routine practice in orthopedic wards [26, 27], indicating that the incidence of UTIs is expected to continue rising [28, 29]. Investigating the connection between admission hypoalbuminemia and UTIs holds significant clinical importance due to its substantial impact on infection prevention.

Current scientific literature indicates that hip fracture surgery, in contrast to elective surgery, cannot significantly improve the preoperative nutritional status by delaying the procedure. Nonetheless, early postoperative nutritional intervention holds the potential to enhance surgical outcomes and reduce complications [13, 14, 30, 31]. Hence, our aim is to further investigate the correlation between hypoalbuminemia and UTIs in hospitalized patients. This investigation will provide additional evidence to support preoperative risk assessment and postoperative nutritional intervention strategies, ultimately leading to a better prognosis for elderly patients with hip fractures.

## Materials and methods

The study has received approval from the Institutional Review Board (IRB) for all aspects of the research. Only clinical data was collected, ensuring the exclusion of personal or identifiable information. Therefore, considering the study's design and data characteristics, the necessity of obtaining informed consent was waived by the IRB. This retrospective analysis utilized electronic medical records from our institution, focusing on elderly patients (aged 60 years and above) admitted between March 2013 and March 2023 with acute hip fractures.

The inclusion criteria comprised patients who underwent joint replacement or orthopedic surgery for hip fractures. Exclusion criteria included multiple fractures, old or pathological fractures, conservative treatment, revision or reoperation for any reason, long-term use of immunosuppressive agents such as glucocorticoids, previous infectious complications, antibiotic treatment at admission, pre-existing or diagnosed urinary tract infections on admission, absence of laboratory tests like urine culture or analysis during hospitalization, lack of serum albumin level measurement within 24 h of admission, in-hospital mortality, or incomplete data.

### Exposure

Blood samples were collected from hip fracture patients within 24 h of admission to determine the presence of baseline hypoalbuminemia. Hypoalbuminemia is defined as a total protein level < 35 g/L [32, 33], while normal albumin levels are defined as total albumin levels ≥ 35 g/L. To examine the relationship between dose and response [34, 35], albumin levels were categorized as mild hypoalbuminemia (34.9–30 g/L), moderate hypoalbuminemia (29.9–25 g/L), and severe hypoalbuminemia (≤ 24.9 g/L). Additionally, the albumin levels of the patients upon admission were divided into four groups based on quartiles: the first group (*Q*1: 41.0–52.0 g/L), the second group (*Q*2: 38.0–40.9 g/L), the third group (*Q*3: 35.0–37.9 g/L), and the fourth group (*Q*4: 15.3–34.9 g/L).

### Outcome

The primary outcome of this study was postoperative urinary tract infections (UTIs). Upon initial admission to the orthopedic ward, urine tests and cultures were routinely collected from the patients. Following orthopedic surgical treatment, regular urine cultures were conducted every 3 days (specifically on Tuesdays and Fridays). To ensure standardized urine collection, clinical nurses in the orthopedic department received regular training from the Hospital Infection Control Committee. This training emphasized the use of aseptic techniques and standard disinfection methods for collecting urine samples, which were promptly sent to the microbiology laboratory.

According to the guidelines provided by the Centers for Disease Control and Prevention in the United States [36], UTIs were defined if patients met the following criteria: (1) presence of fever (> 38 degrees Celsius or 100.4 degrees Fahrenheit), urinary urgency, urinary frequency, dysuria, suprapubic tenderness; (2) positive urine culture indicating bacteriuria (> 10^5^ CFU/mL) or positive urine analysis results, such as the presence of leukocyte esterase and nitrites in mid-stream urine specimens.

The assessment of infection events was independently performed by three members (WY, WW, and WYT), with any discrepancies ultimately resolved by senior researchers (QML and WBD). Lastly, patients diagnosed with UTIs received routine antibiotic treatment.

### Covariables

Data were collected by extracting information from the hospital health information system. Prior to identifying potential risk factors, a comprehensive literature review was conducted, and group meetings were held with doctors and nurses [37–39]. Three trained researchers (WY, WW, and WYT) extracted and compiled data from electronic medical records including (1) demographic information (age, gender, smoking, and drinking status); (2) comorbidities (hypertension, diabetes, coronary heart disease, stroke, chronic kidney disease, bladder urinary tract disease, benign prostatic hyperplasia, urinary tract stones, and history of tumors); (3) surgery-related indicators (type of hip fracture, American Society of Anesthesiologists (ASA) classification, surgical approach, indwelling urinary catheterization, duration of indwelling urinary catheter, surgical time, patient bedridden time); and (4) laboratory-related indicators (red blood cell count, white blood cell count, neutrophil count, lymphocyte count, blood urea nitrogen, creatinine, uric acid, and blood glucose levels). In cases where multiple laboratory measurements were available prior to surgery, the measurement closest to the admission time was selected for analysis. To ensure unbiased data extraction, all data underwent a secondary review by senior researchers (QML and WBD).

### Statistical analysis

Categorical variables were presented as counts (%), while continuous variables were expressed as mean ± standard deviation. The Mantel–Haenszel chi-square test or analysis of variance was conducted to compare trends between groups. Two-sided *p* values of less than 0.05 were considered statistically significant.

To address potential confounding effects resulting from intergroup distribution differences, propensity score matching analysis was performed on all covariates [40]. A 1:1 matching was carried out using a nearest-neighbor matching algorithm, with a caliper width set at 0.25 standard deviations, between the low albuminemia group and the normal albuminemia group. Following matching, the standardized mean difference (SMD) of all covariates was calculated to assess balance before and after propensity score matching (PSM), with a value ≥ 0.10 indicating imbalance. Binary logistic regression analysis was performed using the matched cohort to obtain PSM-adjusted odds ratios (ORs) and 95% confidence intervals (CIs).

For examining the association between serum albumin levels and UTIs, logistic regression analysis was employed. In the univariate logistic regression analysis, potential confounding factors with *p* values ≥ 0.1 were adjusted, while variables with *p* < 0.10 were included in the subsequent multivariate logistic regression analysis. To assess the robustness of the association between albumin levels and UTIs, sensitivity analysis was performed using quartiles, and optimal thresholds was determined by dose–response relationship analysis. Additionally, the *E* value (https://www.evalue-calculator.com/evalue/) was utilized to evaluate the potential impact of unmeasured confounding factors on this relationship [41].

Regarding the further exploration of the relationship between low albuminemia and UTIs, a subgroup analysis was conducted on the propensity score-matched (PSM) cohort. The PSM cohort was divided into multiple groups based on all covariates, and univariate logistic regression analysis was performed to calculate the OR and 95% CI of UTIs associated with low albuminemia. The relationship between the subgroups was assessed with statistical significance defined as a p-value of less than 0.01 to account for multiple subgroups [42]. Statistical analyses were carried out using GraphPad Prism 9.0 and R version 4.2.0.

## Results

**Fig. 1:**
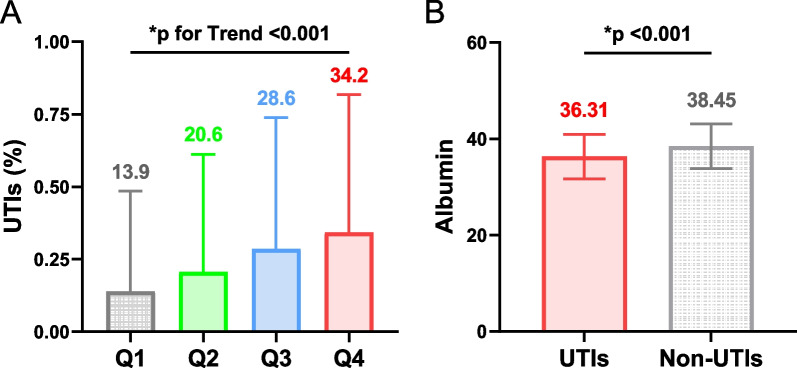
Bar graphs demonstrate the correlation between urinary tract infections and serum albumin. **A** Prevalence of urinary tract infections in patients at the serum albumin quartile distribution (*p* for Trend < 0.001). **B** Serum albumin levels were higher in the UTIs group than in the non-UTIs group (*p* < 0.001). UTIs refer to urinary tract infections; Non-UTIs refer to non-urinary tract infections

A total of 1279 elderly patients who met the criteria for hip fractures were included in the study (see Additional file 1: Fig. S1). Among them, 298 patients (23.3%) developed postoperative UTIs. Table 1 presents the baseline characteristics of the patients, categorized by the severity of admission albumin levels. The mean age of the included elderly patients with hip fractures was 74.7 years and 60.3% of them were female. Participants with low albumin levels upon admission were older, had a higher prevalence of comorbidities, and were at a higher risk of intertrochanteric and subtrochanteric fractures of the femur. They also exhibited higher preoperative ASA grades, longer durations of urinary catheterization and bed rest, as well as relatively lower levels of red blood cells, lymphocytes, and blood urea nitrogen in laboratory tests. Furthermore, as admission albumin levels decreased, there was a gradual increase in the incidence of postoperative UTIs (*p* for trend < 0.001; Fig. 1A). The UTIs group had significantly lower admission albumin levels compared to the non-UTIs group (*p* < 0.001; Fig. 1B).

**Table 1:** Baseline characteristics of the patients by albumin levels (g/L)

Characteristics	Total patient (*n* = 1279)	Albumin level quartile (g/L)				*P* for trend†
		*Q*1 (41.0–52.0; *n* = 374)	*Q*2 (38.0–40.9; *n* = 344)	*Q*3 (35.0–37.9; *n* = 301)	*Q*4 (15.3–34.9; *n* = 260)	
*Demographic*						
Age, × years (Mean, SD)	74.70 (9.55)	69.94 (8.27)	73.45 (9.15)	77.85 (8.25)	79.58 (9.56)	< 0.001
Female gender (*n*, %)	771 (60.28%)	220 (58.82%)	215 (62.50%)	187 (62.13%)	149 (57.31%)	0.818
Smoking (*n*, %)	218 (17.04%)	77 (20.59%)	65 (18.90%)	45 (14.95%)	31 (11.92%)	0.002
Alcohol (*n*, %)	148 (11.57%)	54 (14.44%)	46 (13.37%)	26 (8.64%)	22 (8.46%)	0.005
*Comorbidities*						
Hypertension (*n*, %)	641 (50.12%)	156 (41.71%)	158 (45.93%)	187 (62.13%)	140 (53.85%)	< 0.001
Diabetes (*n*, %)	297 (23.22%)	82 (21.93%)	74 (21.51%)	89 (29.57%)	52 (20.00%)	0.666
Cardiovascular disease (*n*, %)	394 (30.81%)	78 (20.86%)	101 (29.36%)	119 (39.53%)	96 (36.92%)	< 0.001
Stroke (*n*, %)	332 (25.96%)	68 (18.18%)	81 (23.55%)	91 (30.23%)	92 (35.38%)	< 0.001
Chronic kidney disease (*n*, %)	65 (5.08%)	11 (2.94%)	16 (4.65%)	22 (7.31%)	16 (6.15%)	0.019
Vesicoureteral disease (*n*, %)	61 (4.77%)	11 (2.94%)	13 (3.78%)	22 (7.31%)	15 (5.77%)	0.019
Prostate hyperplasia (*n*, %)	30 (2.35%)	5 (1.34%)	11 (3.20%)	5 (1.66%)	9 (3.46%)	0.209
Urolithiasis (*n*, %)	21 (1.64%)	3 (0.80%)	7 (2.03%)	7 (2.33%)	4 (1.54%)	0.353
Neoplasms (*n*, %)	121 (9.46%)	37 (9.89%)	27 (7.85%)	36 (11.96%)	21 (8.08%)	0.911
*Operation*						
Fracture type						
Femoral neck fracture (*n*, %)	684 (53.48%)	258 (68.98%)	204 (59.30%)	129 (42.86%)	93 (35.77%)	< 0.001
Intertrochanteric fracture (*n*, %)	521 (40.73%)	93 (24.87%)	124 (36.05%)	156 (51.83%)	148 (56.92%)	
Subtrochanteric fracture (*n*, %)	74 (5.79%)	23 (6.15%)	16 (4.65%)	16 (5.32%)	19 (7.31%)	
*ASA*						
III–IV (*n*, %)	712 (55.67%)	156 (41.71%)	177 (51.45%)	197 (65.45%)	182 (70.00%)	< 0.001
I–II (*n*, %)	567 (44.33%)	218 (58.29%)	167 (48.55%)	104 (34.55%)	78 (30.00%)	
*Surgery method*						
Total hip arthroplasty (*n*, %)	162 (12.67%)	66 (17.65%)	49 (14.24%)	23 (7.64%)	24 (9.23%)	0.113
Hemiarthroplasty (*n*, %)	322 (25.18%)	86 (22.99%)	99 (28.78%)	83 (27.57%)	54 (20.77%)	
Intramedullary nail fixation (*n*, %)	416 (32.52%)	80 (21.39%)	94 (27.33%)	128 (42.52%)	114 (43.85%)	
Internal fixation with steel plate (*n*, %)	170 (13.29%)	36 (9.63%)	42 (12.21%)	43 (14.29%)	49 (18.85%)	
Internal fixation with hollow nails (*n*, %)	209 (16.34%)	106 (28.34%)	60 (17.44%)	24 (7.97%)	19 (7.31%)	
Catheterization (*n*, %)	589 (46.05%)	149 (39.84%)	156 (45.35%)	158 (52.49%)	126 (48.46%)	0.005
Indwelling catheter time, × days (Mean, SD)	1.77 (3.42)	1.18 (2.38)	1.61 (3.07)	1.96 (3.13)	2.58 (4.97)	< 0.001
Intraoperative time, × hours (Mean, SD)	1.66 (0.80)	1.69 (0.88)	1.62 (0.74)	1.60 (0.70)	1.76 (0.85)	0.501
Bedridden time, × days (Mean, SD)	5.89 (4.02)	5.12 (3.37)	5.62 (3.43)	6.41 (4.23)	6.75 (5.01)	< 0.001
*Laboratory findings (Mean, SD)*						
RBC count, × 10^9^/L	3.93 (0.68)	4.32 (0.58)	3.98 (0.59)	3.76 (0.59)	3.49 (0.67)	< 0.001
WBC count, × 10^9^/L	8.85 (2.87)	8.93 (2.72)	8.76 (2.53)	8.92 (3.11)	8.77 (3.23)	0.653
NEU count, × 10^9^/L	6.78 (2.79)	6.69 (2.72)	6.71 (2.45)	6.93 (2.89)	6.84 (3.18)	0.322
LYM count, × 10^9^/L	1.33 (0.67)	1.50 (0.63)	1.31 (0.64)	1.25 (0.77)	1.21 (0.59)	< 0.001
BUN, × mmol/L	7.40 (4.83)	6.51 (3.27)	6.90 (3.27)	8.44 (7.51)	8.11 (4.14)	< 0.001
Cr, × μmol/L	72.39 (64.22)	65.14 (24.82)	70.55 (68.47)	82.09 (96.23)	74.03 (48.30)	0.010
UA, × μmol/L	288.90 (104.45)	303.48 (111.57)	279.40 (93.47)	290.33 (100.09)	278.84 (110.48)	0.013
Glucose, × mmol/L	6.95 (2.76)	6.79 (2.67)	6.87 (2.78)	7.41 (3.11)	6.78 (2.38)	0.322

*ASA* the American Society of Anesthesiologists Physical Status Classification System, *RBC* red blood cell, *WBC* white blood cell, *NEU* neutrophil, *LYM* lymphocyte, *BUN* blood urea nitrogen, *Cr* creatinine, *UA* uric acid

^†^*P* values for linear trend for continuous variables are from the weighted linear regression model, and categorical variables are from the Mantel–Haenszel chi-square test

The patient characteristics before and after propensity score matching are presented in Additional file 1: Table S1. Prior to matching, the average age of the hypoalbuminemia group and the normal albuminemia group were 79.6 years and 73.5 years, respectively (SMD = 0.65). After matching, all covariates were balanced between the two groups (SMD < 0.1).

Following adjustment for covariates in a multivariable logistic regression analysis (see Additional file 1: Table S2), it was found that patients with hypoalbuminemia had a significantly higher incidence of postoperative UTIs compared to those with normal albumin levels (OR 1.86, 95% CI 1.28–2.70). This association remained significant in the propensity score matching analysis (OR 1.87, 95% CI 1.24–2.83; Table 2).

**Table 2:** Comparison of the unadjusted and risk-adjusted outcome by admission albumin levels (normal ≥ 35 g/L vs. low < 35 g/L)

Outcome	Albumin (g/L) [Events, n (%)]	*P*	Unadjusted OR (95% CI)	*P*	Multivariable regression adjusted OR (95% CI)	*P*	PSM adjusted OR (95% CI)	*P*
Urinary tract infections	≥ 35 [209 (20.5)]	< 0.001	1 [Reference]	< 0.001	1 [Reference]	0.001	1 [Reference]	0.003
	< 35 [89 (34.2)]		2.02 (1.50–2.72)		1.86 (1.28–2.70)		1.87(1.24–2.83)	

*CI* confidence interval, *OR* odds ratio, *PSM* propensity scores matching

A correlation was observed between serum albumin levels upon admission and the occurrence of UTIs (Table 3). Following propensity score matching, patients with albumin levels ranging from 34.9 to 30 g/L (OR 1.52, 95% CI 0.99–2.34), 29.9 to 25 g/L (OR 1.95, 95% CI 1.01–3.76), and ≤ 24.9 g/L (OR 1.95, 95% CI 0.45–8.42) exhibited a significantly higher incidence of UTIs compared to patients with albumin levels ≥ 35 g/L (p for trend < 0.02). The trend of increased UTIs incidence among patients with lower albumin levels was further supported by sensitivity analysis using quartiles (*p* for trend < 0.001), with a significant increase observed in *Q*2, *Q*3, and *Q*4. The association between hypoalbuminemia upon admission and UTIs had *E*-values of 2.07, which suggests that unmeasured confounding factors are unlikely to explain the observed findings.

**Table 3:** Unadjusted and adjusted association between admission albumin levels and urinary tract infections (UTIs)

	Albumin (g/L)	Events, n (%)	Unadjusted OR (95% CI)	*P*	Multivariable regression adjusted OR (95% CI)	*P*	PSM adjusted OR (95% CI)	*P*
Continuous	Per 1	NA	1.10 (1.07–1.13)	< 0.001	1.08 (1.04–1.12)	< 0.001	NA	NA
Clinical threshold	≥ 35	209 (20.5)	1 [Reference]	< 0.001*	1 [Reference]	< 0.01*	1 [Reference]	< 0.02*
	34.9–30	63 (32.6)	1.88 (1.34–2.63)		1.80 (1.20–2.70)		1.52 (0.99–2.34)	
	29.9–25	23 (39.7)	2.55 (1.47–4.40)		2.60 (1.32–5.09)		1.95 (1.01–3.76)	
	≤ 24.9	3 (33.3)	1.94 (0.48–7.81)		0.63 (0.13–3.08)		1.95 (0.45–8.42)	
Quartile	Q1 (41.0–52.0)	52 (13.9)	1 [Reference]	< 0.001*	1 [Reference]	< 0.001*	1 [Reference]	< 0.001*
	Q2 (38.0–40.9)	71 (20.6)	1.61 (1.09–2.38)		1.38 (0.88–2.17)		1.26 (0.85–1.86)	
	Q3 (35.0–37.9)	86 (28.6)	2.48 (1.69–3.64)		1.69 (1.06–2.71)		1.79 (1.16–2.77)	
	Q4 (15.3–34.9)	89 (34.2)	3.22 (2.18–4.76)		2.67 (1.61–4.43)		2.74 (1.72–4.38)	

Multivariate logistic regression analyses adjusted for variables with *p* value < 0.10 in univariate regression analyses: Age, Female gender, Smoking, Alcohol, Hypertension, Diabetes, Cardiovascular disease, Stroke, Chronic kidney disease, Vesicoureteral disease, Prostate hyperplasia, Urolithiasis, Fracture type, ASA grade, Surgery method, Catheterization, Indwelling catheter time, Intraoperative time, Bedridden time, RBC count, WBC count, NEU count, LYM count, BUN, Glu, and ALB

*CI* confidence interval, *OR* odds ratio, *PSM* propensity scores matching

*p for trend

**Fig. 2:**
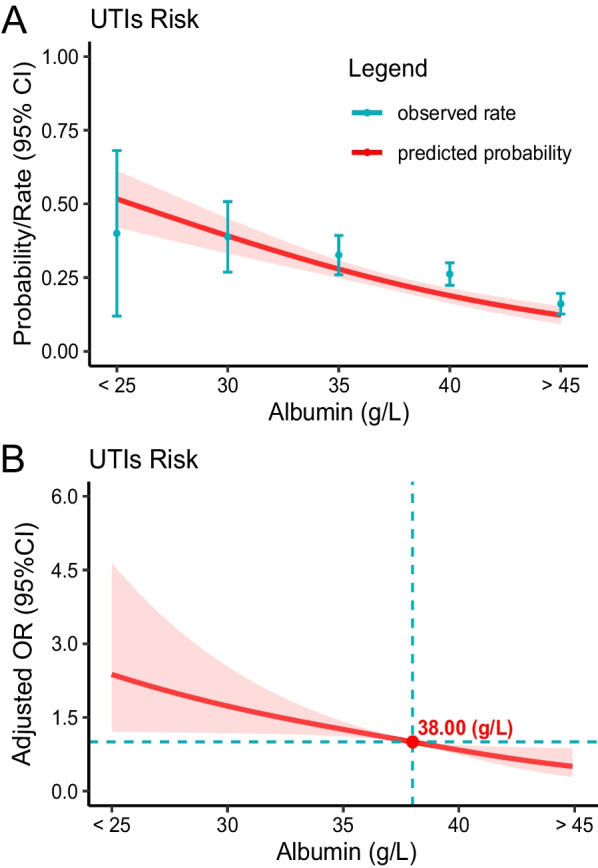
Relationship between admission albumin levels and UTIs in patients with hip fracture. **A** Predicted probabilities and the observed rate of UTIs; **B** Adjusted odds ratios (ORs) and 95% confidence intervals (CIs) are shown for each 5 g/L deviation away from the reference value (35 g/L)

There was a clear dose–response relationship observed between serum albumin levels upon admission and the occurrence of UTIs (Fig. 2). Higher levels of albumin were associated with a decreased predicted probability and incidence rate of UTIs (Fig. 2A). This protective effect was particularly noticeable when albumin levels exceeded 38.00 g/L (Fig. 2B). Additionally, even when albumin levels were considered as a continuous variable, lower levels remained positively correlated with increased odds of UTIs. Specifically, for every 1 g/L decrease in albumin level, the adjusted odds ratio for UTIs was 1.08 (95% CI 1.04–1.12).

**Fig. 3:**
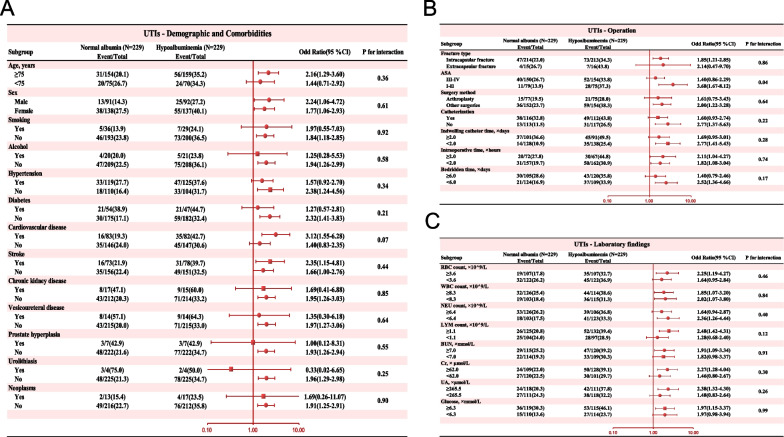
Subgroup analysis of association admission Hypoalbuminemia and UTIs after propensity score matching. **A** Subgroup analysis of variables related to demographic and comorbidities; **B** Subgroup analysis of variables related to operation; **C** Subgroup analysis of variables related to laboratory findings. CI, confidence interval; OR, odds ratio; ASA, the American Society of Anesthesiologists Physical Status Classification System; RBC, red blood cell; WBC, white blood cell; NEU, neutrophil; LYM, lymphocyte; BUN, blood urea nitrogen; Cr, creatinine; UA, uric acid

To further enhance our understanding of the relationship between hypoalbuminemia upon admission and various other variables, we conducted detailed subgroup analyses (Fig. 3). The results revealed that there were no significant interactions between hypoalbuminemia and any of the covariates examined (all *p* values for interactions > 0.01). This suggests that the effects of hypoalbuminemia on the Postoperative UTIs of interest were consistent across different levels of these covariates. The absence of significant interactions implies that the influence of hypoalbuminemia upon admission on Postoperative UTIs remained largely unchanged, regardless of various factors such as age, comorbidities, or surgical procedures.

## Discussion

Previous literature has explored the association between serum albumin levels and UTIs (see Additional file 1: Table S3). However, it is essential to note that these studies were conducted on different populations and utilized varied definitions of serum albumin [22, 23, 25, 43–46]. Therefore, it would be inappropriate to blindly extrapolate their findings to our target population of elderly hip fracture patients. Our study found a clear and significant association between the presence of hypoalbuminemia upon admission and increased odds of developing UTIs in elderly hip fracture patients and a dose–response relationship was observed, as lower albumin levels were associated with a higher risk of infection.

To our knowledge, no previous studies have specifically examined the association between serum albumin levels and UTIs in elderly patients with hip fractures. However, recent research has increasingly recognized the importance of serum albumin in this patient population and its correlation with perioperative complications and long-term mortality after surgery [12, 16, 17, 47–52]. For instance, Tian et al. [47] conducted a systematic review of 3,147 elderly patients with hip fractures and found that preoperative hypoalbuminemia was independently associated with the occurrence of postoperative pneumonia (OR 6.18, 95% CI 3.15–11.98). Kishawi et al. [17] analyzed a national database and discovered that patients with low preoperative albumin levels had a significantly higher risk of postoperative infections and other adverse outcomes in primary total joint replacement surgeries. Yang et al. [49] conducted a follow-up study involving 328 patients with hip fractures and revealed a correlation between albumin levels and postoperative delirium (*p* < 0.001). Additionally, a meta-analysis by Wang et al. [50] demonstrated that albumin levels were associated with an increased risk of deep vein thrombosis (OR 1.42, 95% CI 1.10–1.82). Panteli et al. [51] conducted an 8-years retrospective study in a level I trauma center, investigating all hip fracture patients, and observed a significant association between hypoalbuminemia upon admission and 1-year postoperative mortality (OR 4.82, 95% CI 2.08–11.19). Other studies have also affirmed the relationship between hypoalbuminemia and long-term mortality in patients with hip fractures [12, 16, 52].

Various previous studies have also demonstrated a correlation between serum albumin levels and UTIs in elderly patients [11, 23, 44, 45]. Kitano et al. [23] conducted a study involving 286 elderly patients with UTIs and observed a significant correlation between hypoalbuminemia and the occurrence of UTIs. Furthermore, Tal et al. [44] identified a strong association between low serum albumin levels and mortality in elderly patients with UTIs (*p* < 0.002). Similarly, Ryu et al. [45], in their study comprising 1159 elderly patients with UTIs, found a correlation between albumin levels and mortality (OR 0.83, 95% CI 0.81–0.85). Of particular interest, Cabrerizo et al. [11] discovered that low albumin levels < 38 g/L were associated with an increased risk of postoperative complications, specifically infections, in elderly patients with hip fractures. These findings align with the results of our research, which reveals a dose–response relationship indicating a higher incidence of UTIs in elderly patients with hip fractures when serum albumin levels < 38 g/L.

Subgroup analyses also reinforced our main conclusions concerning the association between hypoalbuminemia and the incidence of postoperative UTIs. Our comprehensive subgroup analyses provided insight into the potential moderating effects of covariates on the association between hypoalbuminemia and the outcome. This enhances the reliability, validity, and clinical relevance of our findings, thereby reinforcing the significance of our study. However, subgroup analyses that rely solely on propensity score-matched cohorts might not fully represent the entirety of older patients with hip fracture. Although matching is crucial to reduce confounding and enhance the internal validity of our study, it does impact the generalizability of our findings. Hence, we emphasize the importance of interpreting the results of subgroup analyses cautiously and the necessity for future studies with larger sample sizes to validate and broaden our findings.

The precise mechanism underlying the association between serum albumin levels and UTIs remains unclear. However, recent evidence suggests that in patients with hip fractures, the sharp decline in serum albumin levels may be attributed to inflammation rather than pre-existing malnutrition [11, 14, 18]. Albumin, the most abundant plasma protein, is exclusively synthesized by the liver, and its metabolic functions are not yet fully understood [53]. Apart from its well-established role in maintaining fluid-electrolyte homeostasis, albumin may also have immunomodulatory properties [54]. Circulating albumin interacts with various inflammatory mediators, thereby promoting neutrophil degranulation and enhancing phagocytic activity [55]. Consequently, suboptimal serum albumin levels may impair the efficiency of the immune system, leading to a heightened susceptibility to infectious complications. Additionally, hypoalbuminemia serves as a simple marker of malnutrition, which is a primary cause of compromised immune response and serves as a robust predictor of hospital-acquired infections [56, 57].

Urinary tract infections (UTIs) significantly contribute to an increased incidence and adverse prognosis in fracture patients, particularly those with hip fractures [58, 59]. A retrospective analysis of 93,637 Danish patients with hip fractures revealed that UTIs were a substantial factor associated with elevated mortality rates, specifically in females [58]. Furthermore, Sun et al.'s predictive model demonstrated a correlation between UTIs and increased mortality rates among patients with hip fractures [59]. Therefore, the effective management and prevention of UTIs in elderly patients with hip fractures are of paramount importance for reducing incidence rates, mortality rates, and associated costs.

It is reassuring to note that serum albumin levels not only hold significant implications for predicting adverse events but can also be positively influenced through appropriate nutritional management. Extensive research supports the concept that early nutritional optimization, involving caloric and protein supplementation, can effectively regulate immune function, maintain normal cellular metabolism, and improve patient outcomes [13, 17, 60, 61]. Studies have demonstrated that preoperative nutritional interventions can significantly enhance the prognosis of infection-related complications following fracture surgery [62, 63]. Additionally, preoperative oral nutritional supplementation (ONS) has been shown to prevent complications from worsening in elderly patients undergoing hip fracture surgery [64]. Given these findings, further research is warranted to explore strategies aimed at elevating serum albumin levels to mitigate the risk of adverse outcomes and to investigate other nutritional markers that may influence surgical outcomes. An interesting recent study suggests that using human serum albumin nanoparticles as a multifunctional carrier for targeted antibiotic delivery could potentially enhance therapeutic efficacy in patients with UTIs [65].

## Limitations

There are several limitations that need consideration when interpreting the results of this study. Firstly, being a retrospective analysis conducted at a single center, there is a potential risk of selection bias, thereby limiting the generalizability of our findings. Secondly, this study primarily focused on establishing associations rather than establishing causality. Hence, further prospective studies are required to corroborate our findings. Moreover, due to the limited available inpatient data, we could not analyze the association between admission albumin levels and long-term patient follow-up. Lastly, it is important to acknowledge that serum albumin levels tend to fluctuate during hospitalization. Despite our efforts to minimize confounding effects by only utilizing baseline levels at admission, we did not analyze the changes in albumin levels throughout hospitalization.

## Conclusions

The results of this study indicate a significant association between hypoalbuminemia on admission and the risk of postoperative UTIs in patients with hip fractures. Additionally, a dose–response relationship is observed between serum albumin levels and the occurrence of postoperative UTIs. Therefore, healthcare providers should remain vigilant for UTIs development and promptly intervene when patients exhibit serum albumin levels < 38 g/L upon admission.

### Supplementary Information

**Additional file1**. **Figure S1**: Flow diagram for selection of cohorts. **Table S1**: Patient characteristics before and after propensity score matching by admission albumin levels (low < 35 g/L vs. normal ≥ 35 g/L). **Table S2**: Multivariate Analysis for urinary tract infections. **Table S3**: Literatures on the correlation between albumin level and UTIs.

## Data Availability

All the data used and analyzed during the current study are available from the corresponding author upon reasonable request.
