# Supplementary material for: Correlation between admission hypoalbuminemia and postoperative urinary tract infections in elderly hip fracture patients
Source: J Orthop Surg Res. 2023 Oct 14;18:774. doi: 10.1186/s13018-023-04274-7 (PMC10576304; doi:10.1186/s13018-023-04274-7)
Supplement: Supplementary file 1 — Additional file1. Figure S1: Flow diagram for selection of cohorts. Table S1: Patient characteristics before and after propensity score matching by admission albumin levels (low < 35 g/L vs. normal ≥ 35 g/L). Table S2: Multivariate Analysis for urinary tract infections. Table S3: Literatures on the correlation between albumin level and UTIs. [file 13018_2023_4274_MOESM1_ESM.docx]

**Appendix:**

**eFigure1 Flow diagram for selection of cohorts**

**eTable1 Patient characteristics before and after propensity score matching by admission albumin levels (low < 35 g/L vs. normal ≥ 35 g/L)**

**eTable2 Multivariate Analysis for urinary tract infections**

**eTable3 Literatures on the correlation between albumin level and UTIs**

**
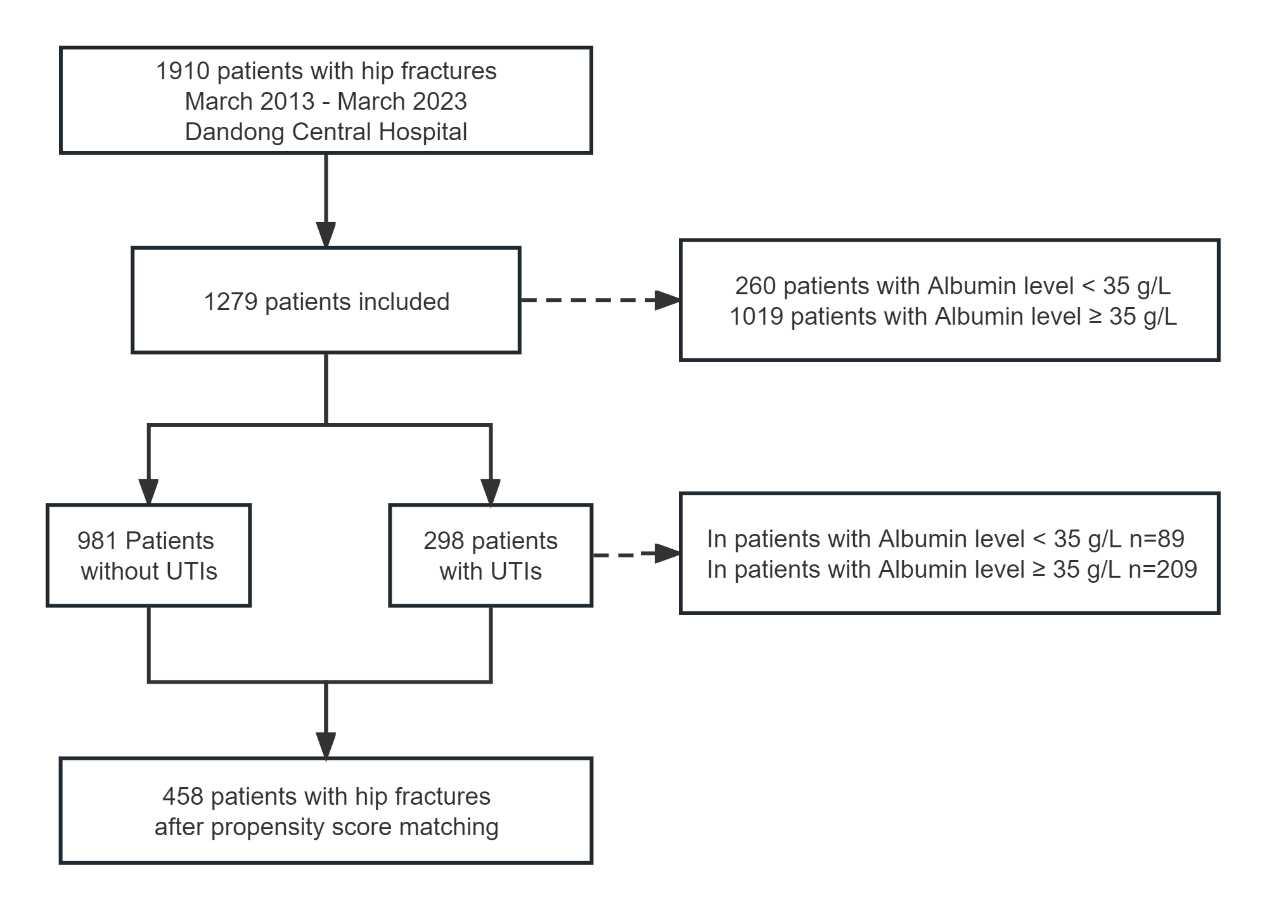
**

**eFigure1 Flow diagram for selection of cohorts**

**eTable1 Patient characteristics before and after propensity score matching by admission albumin levels (low < 35 g/L vs. normal ≥ 35 g/L)**

| Characteristics | Before matching | | | After matching | | |
| --- | --- | --- | --- | --- | --- | --- |
|  | Hypoalbuminemia (n=260) | Normal albumin (n=1019) | SMD | Hypoalbuminemia (n=229) | Normal albumin (n=229) | SMD |
| Demographic |  |  |  |  |  |  |
| Age, × years (Mean, SD) | 79.58 (9.56) | 73.46 (9.14) | 0.65 | 78.58 (9.47) | 78.07 (9.11) | 0.05 |
| Female gender (n, %) | 149 (57.31%) | 622 (61.04%) | 0.08 | 137 (59.83%) | 138 (60.26%) | 0.01 |
| Smoking (n, %) | 31 (11.92%) | 187 (18.35%) | 0.18 | 29 (12.66%) | 36 (15.72%) | 0.09 |
| Alcohol (n, %) | 22 (8.46%) | 126 (12.37%) | 0.13 | 21 (9.17%) | 20 (8.73%) | 0.02 |
| Comorbidities |  |  |  |  |  |  |
| Hypertension (n, %) | 140 (53.85%) | 501 (49.17%) | 0.09 | 125 (54.59%) | 119 (51.97%) | 0.05 |
| Diabetes (n, %) | 52 (20.00%) | 245 (24.04%) | 0.10 | 47 (20.52%) | 54 (23.58%) | 0.07 |
| Cardiovascular disease (n, %) | 96 (36.92%) | 298 (29.24%) | 0.16 | 82 (35.81%) | 83 (36.24%) | 0.01 |
| Stroke (n, %) | 92 (35.38%) | 240 (23.55%) | 0.26 | 78 (34.06%) | 73 (31.88%) | 0.05 |
| Chronic kidney disease (n, %) | 16 (6.15%) | 49 (4.81%) | 0.06 | 15 (6.55%) | 17 (7.42%) | 0.03 |
| Vesicoureteral disease (n, %) | 15 (5.77%) | 46 (4.51%) | 0.06 | 14 (6.11%) | 14 (6.11%) | <0.001 |
| Prostate hyperplasia (n, %) | 9 (3.46%) | 21 (2.06%) | 0.09 | 7 (3.06%) | 7 (3.06%) | <0.001 |
| Urolithiasis (n, %) | 4 (1.54%) | 17 (1.67%) | 0.01 | 4 (1.75%) | 4 (1.75%) | <0.001 |
| Neoplasms (n, %) | 21 (8.08%) | 100 (9.81%) | 0.06 | 17 (7.42%) | 13 (5.68%) | 0.07 |
| Operation |  |  |  |  |  |  |
| Fracture type |  |  |  |  |  |  |
| Femoral neck fracture (n, %) | 93 (35.77%) | 591 (58.00%) | 0.41 | 89 (38.86%) | 89 (38.86%) | 0.01 |
| Intertrochanteric fracture (n, %) | 148 (56.92%) | 373 (36.60%) |  | 124 (54.15%) | 125 (54.59%) |  |
| Subtrochanteric fracture (n, %) | 19 (7.31%) | 55 (5.40%) |  | 16 (6.99%) | 15 (6.55%) |  |
| ASA |  |  |  |  |  |  |
| Ⅲ-Ⅳ (n, %) | 182 (70.00%) | 530 (52.01%) | 0.38 | 154 (67.25%) | 150 (65.50%) | 0.04 |
| Ⅰ-Ⅱ (n, %) | 78 (30.00%) | 489 (47.99%) |  | 75 (32.75%) | 79 (34.50%) |  |
| Surgery method |  |  |  |  |  |  |
| Total Hip Arthroplasty (n, %) | 24 (9.23%) | 138 (13.54%) | 0.01 | 24 (10.48%) | 19 (8.30%) | 0.02 |
| Hemiarthroplasty (n, %) | 54 (20.77%) | 268 (26.30%) |  | 51 (22.27%) | 58 (25.33%) |  |
| Intramedullary nail fixation (n, %) | 114 (43.85%) | 302 (29.64%) |  | 96 (41.92%) | 97 (42.36%) |  |
| Internal fixation with steel plate (n, %) | 49 (18.85%) | 121 (11.87%) |  | 41 (17.90%) | 43 (18.78%) |  |
| Internal fixation with hollow nails (n, %) | 19 (7.31%) | 190 (18.65%) |  | 17 (7.42%) | 12 (5.24%) |  |
| Catheterization (n, %) | 126 (48.46%) | 463 (45.44%) | 0.06 | 112 (48.91%) | 116 (50.66%) | 0.04 |
| Indwelling catheter time, ×days (Mean, SD) | 2.58 (4.97) | 1.56 (2.87) | 0.25 | 2.37 (4.03) | 2.61 (4.16) | 0.06 |
| Intraoperative time, ×hours (Mean, SD) | 1.76 (0.85) | 1.64 (0.79) | 0.15 | 1.72 (0.82) | 1.77 (0.86) | 0.05 |
| Bedridden time, ×days (Mean, SD) | 6.75 (5.01) | 5.67 (3.70) | 0.24 | 6.63 (5.08) | 6.37 (4.69) | 0.05 |
| Laboratory findings (Mean, SD) |  |  |  |  |  |  |
| RBC count, ×10^9/L | 3.49 (0.67) | 4.04 (0.63) | 0.84 | 3.58 (0.62) | 3.54 (0.61) | 0.07 |
| WBC count, ×10^9/L | 8.77 (3.23) | 8.87 (2.78) | 0.03 | 8.71 (3.09) | 8.88 (2.92) | 0.06 |
| NEU count, ×10^9/L | 6.84 (3.18) | 6.77 (2.68) | 0.03 | 6.77 (3.03) | 6.93 (2.70) | 0.05 |
| LYM count, ×10^9/L | 1.21 (0.59) | 1.36 (0.68) | 0.24 | 1.23 (0.61) | 1.24 (0.82) | 0.01 |
| BUN, ×mmol/L | 8.11 (4.14) | 7.21 (4.98) | 0.20 | 8.05 (4.25) | 8.01 (5.49) | 0.01 |
| Cr, ×μmol/L | 74.03 (48.30) | 71.98 (67.69) | 0.04 | 74.41 (50.14) | 77.15 (81.46) | 0.04 |
| UA, ×μmol/L | 278.84 (110.48) | 290.75 (100.22) | 0.12 | 281.10 (113.14) | 284.80 (98.87) | 0.04 |
| Glucose, ×mmol/L | 6.78 (2.38) | 7.00 (2.85) | 0.08 | 6.83 (2.45) | 6.91 (2.19) | 0.04 |

SMD, standardized mean difference, used to evaluate the balance before and after PSM, ≥ 0.1 indicates imbalance.

**eTable2 Multivariate Analysis for urinary tract infections**

| Characteristics | Univariate | | | Multivariate | | |
| --- | --- | --- | --- | --- | --- | --- |
|  | OR | 95%CI | p-value | OR | 95%CI | p-value |
| Demographic |  |  |  |  |  |  |
| Age, × years | 1.05 | 1.03-1.06 | <0.001 | 1.02 | 0.99-1.04 | 0.07 |
| Female gender | 2.20 | 1.65-2.93 | <0.001 | 2.53 | 1.69-3.77 | <0.001 |
| Smoking | 0.56 | 0.38-0.83 | 0.003 | 1.03 | 0.59-1.79 | 0.93 |
| Alcohol | 0.54 | 0.34-0.87 | 0.01 | 1.06 | 0.54-2.09 | 0.86 |
| Comorbidities |  |  |  |  |  |  |
| Hypertension | 1.99 | 1.53-2.60 | <0.001 | 1.35 | 0.97-1.88 | 0.08 |
| Diabetes | 2.94 | 2.21-3.90 | <0.001 | 1.40 | 0.95-2.07 | 0.09 |
| Cardiovascular disease | 1.77 | 1.35-2.32 | <0.001 | 1.19 | 0.86-1.65 | 0.30 |
| Stroke | 1.44 | 1.08-1.91 | 0.01 | 1.01 | 0.72-1.42 | 0.96 |
| Chronic kidney disease | 3.23 | 1.95-5.36 | <0.001 | 2.02 | 1.03-3.95 | 0.04 |
| Vesicoureteral disease | 4.24 | 2.52-7.14 | <0.001 | 2.15 | 1.06-4.35 | 0.03 |
| Prostate hyperplasia | 2.97 | 1.43-6.17 | 0.003 | 4.60 | 1.86-11.35 | 0.001 |
| Urolithiasis | 3.06 | 1.29-7.28 | 0.01 | 0.76 | 0.25-2.36 | 0.64 |
| Neoplasms | 0.89 | 0.57-1.41 | 0.62 | <NA> | <NA> | <NA> |
| Operation |  |  |  |  |  |  |
| Fracture type | 0.64 | 0.52-0.79 | <0.001 | 0.67 | 0.50-0.91 | 0.01 |
| ASA | 1.41 | 1.08-1.84 | 0.01 | 0.64 | 0.46-0.89 | 0.01 |
| Surgery method | 0.89 | 0.80-0.99 | 0.03 | 0.92 | 0.78-1.08 | 0.30 |
| Catheterization | 2.21 | 1.70-2.89 | <0.001 | 1.22 | 0.83-1.80 | 0.31 |
| Indwelling catheter time, ×days | 1.20 | 1.15-1.25 | <0.001 | 1.15 | 1.08-1.21 | <0.001 |
| Intraoperative time, ×hours | 1.16 | 1.00-1.35 | 0.06 | 1.08 | 0.89-1.32 | 0.44 |
| Bedridden time, ×day | 1.08 | 1.04-1.11 | <0.001 | 1.03 | 0.99-1.07 | 0.07 |
| Laboratory findings |  |  |  |  |  |  |
| RBC count, ×10^9/L | 0.67 | 0.55-0.82 | <0.001 | 0.96 | 0.75-1.24 | 0.75 |
| WBC count, ×10^9/L | 1.05 | 1.01-1.10 | 0.02 | 1.05 | 0.83-1.32 | 0.69 |
| NEU count, ×10^9/L | 1.07 | 1.02-1.12 | 0.004 | 0.96 | 0.76-1.21 | 0.70 |
| LYM count, ×10^9/L | 0.74 | 0.59-0.94 | 0.01 | 0.91 | 0.66-1.25 | 0.56 |
| BUN, ×mmol/L | 1.04 | 1.01-1.06 | 0.01 | 0.99 | 0.95-1.03 | 0.58 |
| Cr, ×μmol/L | 1.00 | 0.99-1.00 | 0.27 | <NA> | <NA> | <NA> |
| UA, ×μmol/L | 1.00 | 0.99-1.00 | 0.88 | <NA> | <NA> | <NA> |
| Glu, ×mmol/L | 1.26 | 1.20-1.33 | <0.001 | 1.21 | 1.14-1.29 | <0.001 |
| ALB, ×g/L (Dichotomy) | 2.02 | 1.50-2.72 | <0.001 | 1.86 | 1.28-2.70 | 0.001 |

Note: Multivariate logistic regression analyses adjusted for variables with p-value < 0.10 in univariate regression analyses: Age, Female gender, Smoking, Alcohol, Hypertension, Diabetes, Cardiovascular disease, Stroke, Chronic kidney disease, Vesicoureteral disease, Prostate hyperplasia, Urolithiasis, Fracture type, ASA grade, Surgery method, Catheterization, Indwelling catheter time, Intraoperative time, Bedridden time, RBC count, WBC count, NEU count, LYM count, BUN, Glu, and ALB.

**eTable3 Literatures on the correlation between albumin level and UTIs**

| Study | Country | Study type | Research Results | | References |
| --- | --- | --- | --- | --- | --- |
|  |  |  | OR  (95 CI%) | p-value |  |
| Kitano et al. 2021 [23] | Japan | Cohort study | NA | < 0.01 | Kitano H, Shigemoto N, Koba Y, Hara T, Seiya K, Omori K, Shigemura K, Teishima J, Fujisawa M, Matsubara A, Ohge H (2021) Indwelling catheterization, renal stones, and hydronephrosis are risk factors for symptomatic Staphylococcus aureus-related urinary tract infection. World journal of urology 39:511-516. https://doi.org/10.1007/s00345-020-03223-x |
| Li et al. 2023 [43] | China | Cohort study | 12.10  (2.10-69.90) | < 0.01 | Li Y, Liu Y, Huang Y, Zhang J, Ma Q, Liu X, Chen Q, Yu H, Dong L, Lu G (2023) Development and validation of a user-friendly risk nomogram for the prediction of catheter-associated urinary tract infection in neuro-intensive care patients. Intensive & critical care nursing 74:103329. https://doi.org/10.1016/j.iccn.2022.103329 |
| Tal et al. 2005 [44] | Israel | Cohort study | 3.13  (1.56-6.67) | 0.002 | Tal S, Guller V, Levi S, Bardenstein R, Berger D, Gurevich I, Gurevich A (2005) Profile and prognosis of febrile elderly patients with bacteremic urinary tract infection. The Journal of infection 50:296-305. https://doi.org/10.1016/j.jinf.2004.04.004 |
| Ryu et al. 2020 [45] | Korea | Cohort study | 3.17  (2.31-4.34) | < 0.001 | Ryu S, Oh SK, Cho SU, You Y, Park JS, Min JH, Jeong W, Cho YC, Ahn HJ, Kang C (2020) A novel predictive tool for prognosis in elderly patients with urinary tract infection: Modified PRACTICE. The American journal of emergency medicine 38:2002-2006. https://doi.org/10.1016/j.ajem.2020.06.037 |
| Chin et al. 2011 [25] | Korea | Cohort study | 27.00  (2.00-361.20) | < 0.01 | Chin BS, Kim MS, Han SH, Shin SY, Choi HK, Chae YT, Jin SJ, Baek JH, Choi JY, Song YG, Kim CO, Kim JM (2011) Risk factors of all-cause in-hospital mortality among Korean elderly bacteremic urinary tract infection (UTI) patients. Archives of gerontology and geriatrics 52:e50-55. https://doi.org/10.1016/j.archger.2010.05.011 |
| Kishawi et al. 2020 [17] | USA | Cohort study | 1.68  (1.43-1.97) | < 0.0001 | Kishawi D, Schwarzman G, Mejia A, Hussain AK, Gonzalez MH (2020) Low Preoperative Albumin Levels Predict Adverse Outcomes After Total Joint Arthroplasty. The Journal of bone and joint surgery American volume 102:889-895. https://doi.org/10.2106/jbjs.19.00511 |
| Fang et al. 2022 [21] | USA | Cohort study | 1.36  (1.04-1.80) | 0.03 | Fang CJ, Saadat GH, Butler BA, Bokhari F (2022) The Geriatric Nutritional Risk Index Is an Independent Predictor of Adverse Outcomes for Total Joint Arthroplasty Patients. The Journal of arthroplasty 37:S836-s841. https://doi.org/10.1016/j.arth.2022.01.049 |
| D. Bohl et al. 2016 [22] | USA | Cohort study | 2.50  (1.30-4.80) | 0.005 | Bohl DD, Shen MR, Mayo BC, Massel DH, Long WW, Modi KD, Basques BA, Singh K (2016) Malnutrition Predicts Infectious and Wound Complications Following Posterior Lumbar Spinal Fusion. Spine 41:1693-1699. https://doi.org/10.1097/brs.0000000000001591 |
| O. Ukogu et al. 2018 [46] | USA | Cohort study | 5.93  (2.11-16.68) | 0.001 | Ukogu CO, Jacobs S, Ranson WA, Somani S, Vargas L, Lee NJ, Di Capua J, Kim JS, Vig KS, Cho SK (2018) Preoperative Nutritional Status as a Risk Factor for Major Postoperative Complications Following Anterior Lumbar Interbody Fusion. Global spine journal 8:662-667. https://doi.org/10.1177/2192568218760540 |
